# Comparison of brachial and carotid artery ultrasound for assessing extent of subclinical atherosclerosis in HIV: a prospective cohort study

**DOI:** 10.1186/1742-6405-6-11

**Published:** 2009-06-11

**Authors:** Adefowope Odueyungbo, Marek Smieja, Lehana Thabane, Fiona Smaill, Kevin Gough, John Gill, Todd Anderson, Dawn Elston, Sandy Smith, Joseph Beyene, Eva Lonn

**Affiliations:** 1 Department of Clinical Epidemiology and Biostatistics, McMaster University, Hamilton ON, Canada; 2 Centre for Evaluation of Medicines, St Joseph's Healthcare Hamilton, Hamilton ON, Canada; 3 Biostatistics Unit, Father Sean O'Sullivan Research Centre, St Joseph's Healthcare Hamilton, Hamilton ON, Canada; 4 Department of Pathology and Molecular Medicine, McMaster University, Hamilton ON, Canada; 5 Department of Medicine, McMaster University, Hamilton ON, Ontario, Canada; 6 Department of Medicine, University of Toronto, Toronto ON, Canada; 7 Department of Medicine, University of Calgary, Calgary AB, Canada; 8 Department of Cardiac Sciences and Libin Cardiovascular Institute, University of Calgary, Calgary AB, Canada; 9 Department of Public Health Sciences, University of Toronto, Toronto ON, Canada; 10 Canadian HIV Vascular Study Group, Canada

## Abstract

**Background:**

Non-invasive surrogate measures which are valid and responsive to change are needed to study cardiovascular risks in HIV. We compared the construct validity of two noninvasive arterial measures: carotid intima medial thickness (IMT), which measures anatomic disease; and brachial flow-mediated vasodilation (FMD), a measure of endothelial dysfunction.

**Methods:**

A sample of 257 subjects aged 35 years or older, attending clinics in five Canadian centres, were prospectively recruited into a study of cardiovascular risk among HIV subjects. The relationship between baseline IMT or FMD and traditional vascular risk factors was studied using regression analysis. We analyzed the relationship between progression of IMT or FMD and risk factors using fixed-effects models. We adjusted for use of statin medication and CD4 count in both models.

**Results:**

Baseline IMT was significantly associated with age (p < 0.001), male gender (p = 0.034), current smoking status (p < 0.001), systolic blood pressure (p < 0.001) and total:HDL cholesterol ratio (p = 0.004), but not statin use (p = 0.904) and CD4 count (p = 0.929). IMT progression was significantly associated with age (p < 0.001), male gender (p = 0.0051) and current smoking status (p = 0.011), but not statin use (p = 0.289) and CD4 count (p = 0.927). FMD progression was significantly associated with current statin use (p = 0.019), but not CD4 count (p = 0.84). Neither extent nor progression of FMD was significantly associated with any of the examined vascular risk factors.

**Conclusion:**

IMT correlates better than FMD with established cardiovascular risk factors in this cohort of HIV patients. Standardization of protocols for FMD and IMT will facilitate the comparison of results across studies.

## Background

HIV patients may have a higher risk of developing cardiovascular diseases than the general population [1-3]. This higher risk *may *be attributed to HIV infection or to individual drugs (or drug classes) used in treating the infection [1,4]. In particular, studies have shown that protease inhibitors [4] and nucleoside reverse transcriptase inhibitors such as abacavir and didanosine are associated with increased risk of myocardial infarction in HIV patients [5].

Cardiovascular disease is often characterized by development of atherosclerosis, in which plaque is accumulated on the inside of arterial walls [6]. The reference standard for assessing extent of atherosclerosis is coronary angiography, which is costly, invasive and has occasional complications such as vascular injury [7]. Inexpensive, reproducible, validated, non-invasive measurement of sub-clinical atherosclerosis involves the use of ultrasound (US) methods for imaging the carotid and branchial arteries [8-10]. Summary measures obtained from arterial wall thickness have been used as surrogates of extent, severity and progression of atherosclerosis in numerous studies of cardiovascular health involving diverse patient populations [10]. Examples of such measures include carotid intimal medial thickness (IMT), brachial artery flow-mediated vasodilation (FMD) and plaque area [10,11].

Carotid IMT is a measure of anatomic disease, used to identify and determine the extent of early arterial wall changes or structural vascular abnormalities [10,12-14]. Increased carotid IMT is a strong predictor of acute coronary events [10,14,15], and is significantly associated with established cardiovascular risk factors among various study populations [1,9,10,13,14,16-18].

Brachial FMD is a non-invasive and validated measure of endothelial function [19,20]. The endothelium helps to maintain vascular health by releasing both *paracrine *and *autocrine *factors such as nitric oxide (also called *endothelium-derived relaxing factor*). Nitric oxide (NO) promotes smooth muscle relaxation, inhibition of platelet aggregation and adhesion, vasodilation and increased blood flow [21,22]. Thus, endothelial generation of NO is protective against atherogenesis [22]. A reduction in endothelial release of NO indicates endothelial dysfunction and is regarded as an early evidence of atherosclerosis [21-25]. Individuals with coronary artery disease (CAD) may exhibit impaired brachial FMD responses in the brachial arteries [11,20,26].

Impaired brachial FMD has been shown to be significantly associated with cardiovascular risk factors in some [11,24,27], but not all, studies [13,28]. Also, there are conflicting results regarding the association between brachial FMD and cardiovascular events in various patient populations [20,29].

Non-invasive surrogate measures which are valid and responsive to change are needed to study cardiovascular risks associated with HIV or HIV treatment regimens. There are limited data on the relationship between extent/progression of carotid IMT or brachial FMD and traditional vascular risk factors in HIV patients. Further, the relationship between carotid IMT and brachial FMD has not been well studied in HIV patients. In this study, we compare the validity and responsiveness to change of two ultrasound measures: 12-segment carotid artery IMT and brachial artery FMD in Canadian HIV vascular study participants. We also investigate the relationship between these two measures.

## Methods

### Study design and study population

HIV patients aged 35 years or older, attending university-affiliated clinics in five Canadian centers (Hamilton, Toronto, Calgary, Quebec City and Vancouver) are being recruited into an ongoing five-year, prospective, multi-center cohort study to evaluate the association between atherosclerotic progression, anti-retroviral drug regimen, immune reconstitution and standard cardiovascular risk factors. Subjects are recruited regardless of cardiovascular risk factors or past cardiac history. The study was approved by research ethics boards of each study site, and informed consent was obtained from all participants.

**Figure 1:**
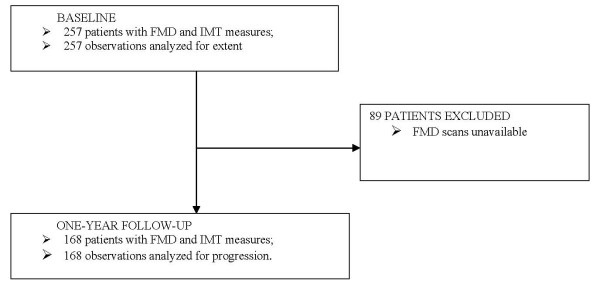
**Flowchart of patients**.

All participants provide a medical history and undergo yearly high-resolution ultrasound using a standardized protocol and centralized reading. As of March 2008, 257 subjects had baseline measurements for carotid IMT and brachial FMD, with 168 patients having one-year follow-up assessments. Measurement of carotid IMT is ongoing, but brachial FMD was discontinued after one-year follow-up due to cost considerations. For this ancillary study, two datasets were created namely: (1) cross-sectional data consisting of 257 patients with baseline carotid IMT and brachial FMD; and (2) progression data consisting of 168 patients with baseline and follow-up measurements for carotid IMT and brachial FMD (Figure 1).

### Clinical characteristics

Data on demographic and certain clinical characteristics of subjects were collected at each centre using questionnaires administered by research staff, or by chart review. Blood pressure was measured twice using a mercury sphygmomanometer, and results averaged. Lipids (total and HDL cholesterol and triglycerides) were measured after overnight fast. LDL-cholesterol concentration was calculated by the Friedewald formula. CD4-T-lymphocyte counts were obtained by FACS analysis performed by the Hamilton Regional Laboratory Medicine Program, and plasma HIV viral load were measured by Chiron bDNA assay at the Central Public Health Laboratory in Toronto, Ontario.

### Ultrasound methods

Ultrasound imaging and readings are conducted by trained personnel using high resolution B-mode ultrasonography, standardized protocol and centralized reading. The ultrasound laboratory in each study site uses imaging systems equipped with 7.5 to 10 MHz linear phase-arrayed vascular transducers. The same imaging system is used for all ultrasound imaging within each center. Ultrasound measurements are recorded on S-VHS tapes, which are later digitized and analyzed offline at the Core Carotid Ultrasound Laboratory (Hamilton, Ontario) by a certified reader blinded to patients' clinical information.

Patients were advised to fast and abstain from caffeine/vasoactive medications 12 hours prior to measurement, and were advised to avoid cigarette smoking (second-hand inclusive) at least four hours prior to imaging. Imaging for carotid IMT was done before brachial FMD on the same day.

#### (A) 12-segment carotid intimal medial thickness (IMT)

Carotid IMT identifies and quantitates early arterial wall changes or structural vascular abnormalities [10,12,13]. A rigorously-standardized, reliable, validated method of '12-segment carotid IMT' developed by Lonn et al [8,30] was used to assess the global extent of atherosclerosis in patients. Images of six well-defined segments (near and far wall of the common carotid, the bifurcation and the internal carotid) were obtained in each of the left and right carotid arteries using high resolution B-mode ultrasonography.

Ultrasound measurements were recorded on S-VHS tapes, which were later digitized and analyzed using the Image-Pro V4.5.1 software (Glen Burnie, Maryland). For each segment a minimum of three frames were measured. The maximum of all measurements from each segment were summed-up and divided by 12 to obtain the "12-segment mean-maximal carotid IMT" [8]. Twelve-segment mean-maximal carotid IMT is higher in individuals with CAD [8,30].

#### (B) Brachial flow-mediated vasodilation (FMD)

Brachial FMD was measured using an extensively validated and reliable method [13,31-33]. End-diastolic ultrasound images of the brachial artery diameter (longitudinally and slightly above the *antebrachial fossa *or upper arm) were obtained at rest and during vasodilator response induced by passive hyperemia (endothelium-dependent dilation).

Each patient rested in a quiet room for 10 minutes, after which sequential images of the brachial artery were obtained within a 45 second interval. Subsequently, a blood pressure cuff was inflated around the right lower arm to at least 200 mm Hg, resulting in occlusion of blood flow to the upper arm. The cuff was released after five minutes, resulting in a marked increase in blood flow due to resistance vessel dilation. The increase in blood flow stimulates the release of NO which mediates the dilation of conduit vessels. Peak brachial artery dilation occurs approximately one minute after cuff release [26]. Another set of sequential images was obtained during peak dilation.

The ultrasound image frames obtained were recorded on S-VHS tapes, from which brachial artery diameters were calculated using Dynamic Endothelial Assessment (DEA) software (Montreal, Quebec). Average diameter of brachial artery (before and after dilation) was obtained from nine sequential images taken at rest and 12 taken during peak artery dilation. Percent flow mediated dilation was expressed as



Conduit vessel dilation is attenuated (smaller %FMD) in individuals with CAD [26].

Twelve-segment carotid IMT and brachial FMD have been standardized and validated in previous studies at the Core Carotid Ultrasound Laboratory (Hamilton, Ontario), with intraclass correlation > 90% and coefficient of variation < 5% for repeat examinations [13,30].

### Statistical analysis

Continuous variables are expressed as mean (standard deviation), while categorical variables are expressed as count (percent) unless otherwise stated.

We hypothesized that "brachial FMD and carotid IMT should correlate well with traditional vascular risk factors for them to be considered good measures of extent, severity or progression of atherosclerosis". This formed the basis for assessment of construct validity. Multiple linear regression models were used to examine the association between baseline carotid IMT or brachial FMD and the well-validated traditional "Framingham" cardiovascular risk factors of age, male gender, current smoking status, systolic blood pressure (SBP) and total:HDL cholesterol ratio using the cross-sectional data. Goodness-of-fit was evaluated by plotting the residuals from models to assess the normality assumption. The distribution of residuals should approximate the normal distribution for good model fit. We also used the co-efficient of determination (R^2^) to quantify the proportion of variation in the dependent variable explained by the independent variables included in the multiple regression models [34].

Fixed effects models were used to study the relationship between progression of carotid IMT or brachial FMD and known cardiovascular risk factors using the progression data. Fixed effects models are useful for longitudinal data in which changes in time-varying covariates such as age, total:HDL cholesterol and SBP may affect the repeated outcome of interest [35]. There is no reason to assume that these quantities are constant over time. Further, the correlation between baseline and follow-up response is incorporated into model specification by assuming a plausible correlation structure. We assumed a "continuous time" version of the auto-regressive (AR(1)) correlation structure (available only for mixed/fixed effects models in SAS^© ^software), to adjust for irregularities in follow-up times [36]. The reason is that many scheduled follow-up visits were not feasible due to circumstances beyond the control of investigators, thus resulting in differential follow-up times for patients. A *time *variable was created by designating the first visit for each patient as (*t*_1 _= 1) and follow-up visits as



The time component is closer to reality by making it a continuous, rather than a discrete, variable. Model fit was assessed using the "Null Model Likelihood Ratio Test" [37]. The "Null Model Likelihood Ratio Test" is a likelihood ratio test of whether the model with a *specified *covariance structure fits better than a model where repeated responses are assumed *independent*. An independent covariance structure is often implausible for repeated measures data. A p-value < 0.05 for the likelihood ratio test shows that the fitted model is better than an *independent *covariance structure model [37]. Model adequacy was also evaluated using Akaike's Information Criterion (AIC) to compare between "continuous time" and "fixed time" AR(1) structures. A smaller AIC indicates better fit [37].

We evaluated the nature of the relationship between baseline carotid IMT and brachial FMD using Pearson correlation co-efficient.

Patients were classified as very low, low, medium/high risk if individual Framingham risk scores were < 5%, 5–9% and ≥ 10% respectively [38]. The medium and high risk categories were combined due to limited numbers of subjects in these categories. Framingham risk scores quantify the 10-year risk of developing "hard" coronary heart disease including myocardial infarction and coronary death [38]. Framingham risk score is a strong predictor of coronary heart disease [38]. One-way analysis of variance (ANOVA) models were used to cross-sectionally examine differences in brachial FMD or carotid IMT by Framingham risk group classification.

We adjusted for current use of statin medication and CD4 count in each regression model. All statistical tests were conducted at 5% significance level. Graphs and analysis results were obtained using SPSS Version 15.0 (SPSS Inc., Chicago, Illinois, USA) and SAS Version 9.1 (SAS Institute Inc., Cary, NC, USA).

The authors had full access to the data and take responsibility for its integrity. All authors have read and agree to the manuscript as written.

## Results

### Baseline and follow-up characteristics

#### Cross-sectional data

**Figure 2:**
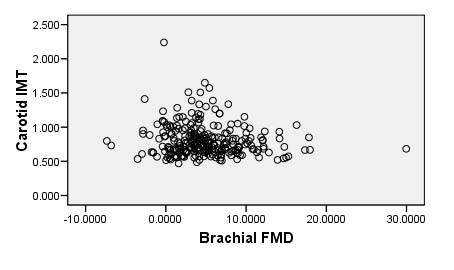
**Carotid IMT versus brachial FMD at baseline**.

There were 257 patients in the baseline extent data with 232(90.3%) males and 25(9.7%) females. Carotid IMT ranged from 0.47 mm to 2.24 mm, with mean(SD) of 0.81(0.23) mm. Brachial FMD ranged from -7.36% to 29.96%, with mean(SD) of 4.95(4.50)%. We found a weak inverse relationship between carotid IMT and brachial FMD at baseline (r = -0.126; p = 0.043; see Figure 2). Other patient characteristics are listed in Table 1.

**Table 1:** Baseline characteristics for extent data (n = 257)

**Variable**	**Estimate**
Male*	232 (90.3)

Age (years)^#^	46.48 (7.86)

Carotid Artery Intima Media Thickness (IMT, mm)^#^	0.81 (0.23)

Flow Mediated Vasodilation (FMD, %)^#^	4.95 (4.50)

Total:HDL Cholesterol^#^	5.28 (1.33)

Systolic Blood Pressure (mm Hg)^#^	120.5 (15.6)

Current Smoking Status* 1	96 (37.5)

Current STATIN use* 1	18 (7.0)

CD4 Count^#^	479.9 (270.6)

Log_10 _Viral Load^#^	2.2 (1.2)

NB) 1 = current smoker/user; * = count(%); # = mean(standard deviation)

Stratifying by Framingham risk group, *dose-response *relationships were found between risk group classification and carotid IMT or brachial FMD (Table 2). Carotid IMT differed significantly between risk groups from ANOVA analysis (p < 0.001). Brachial FMD did not differ significantly across the risk groups from ANOVA results (p = 0.227).

**Table 2:** Baseline characteristics for extent data by Framingham risk group

**Risk group**	**Number of subjects**	**IMT (mm)**	**FMD (%)**
Very low (< 5%)	88	0.68 (0.13)	5.58 (5.45)

Low (5 to 9%)	64	0.78 (0.16)	4.86 (3.59)

Medium/High (10% and above)	105	0.93 (0.27)	4.47 (4.08)

NB) Entries for IMT and FMD are reported as mean (standard deviation); IMT increases significantly with increasing Framingham risk (p < 0.001)

Of the 257 patients assessed at baseline, information on anti-retroviral therapy was available for 253 individuals. There were 85 (34%) patients who were currently on Abacavair, 106 (42%) were on Zidovudine, 61 (24%) on Stavudine, 21 (8%) on Didanosine, 98 (39%) on Efavirenz, 21 (8%) on Nelfinavir and 21 (8%) on Nevirapine. However, we did not test the effects of HIV medications on Carotid IMT/brachial FMD as that was not part of our main goal, which was to validate these measures against traditional risk factors.

#### Progression data

There were 168 patients in the progression dataset with 151(89.9%) males and 17(10.1%) females. Median (interquartile range) follow-up time was 1.02 (0.43) years. At baseline, carotid IMT varied from 0.47 mm to 1.57 mm with mean(SD) of 0.82(0.22) mm, while brachial FMD varied from -6.81% to 29.96% with mean(SD) of 5.10(4.58)%. At one-year follow-up, the measures ranged from 0.50 mm to 1.57 mm with mean(SD) of 0.84(0.23) mm and -13.61% to 25.52% with mean(SD) of 4.40(4.96)% respectively. On average, carotid IMT progressed at 0.02(standard error (SE) = 0.01) mm/year while brachial FMD decreased at 0.84(SE = 0.79)%/year. Summary statistics for other variables are listed in Table 3. Summary data for patients excluded from the progression analyses are summarized in Table 4. Patient distribution appears to be comparable in both included and excluded data, except for viral load and current statin use.

**Table 3:** Baseline and follow-up characteristics for progression data (n = 168)

**Variable**	**Baseline**	**Follow-up**
Male*	151 (89.9)	

AGE (years)^#^	47.19 (8.29)	48.25 (8.34)

IMT (mm)^#^	0.82 (0.22)	0.84 (0.23)

FMD (%)^#^	5.10 (4.58)	4.40 (4.96)

SBP (mm Hg)^#^	120.4 (15.7)	121.1 (13.7)

Total: HDL Cholesterol^#^	5.40 (1.39)	5.18 (1.17)

Current smoking status* 1	60 (35.7)	

Current STATIN use* 1	9 (5.4)	

CD4 Count^#^	495.0 (267.6)	571.3 (883.2)

Log_10 _Viral Load^#^	2.0 (1.1)	2.1 (1.2)

NB) 1 = current smoker/user; * = count(%); # = mean(standard deviation)

**Table 4:** Baseline characteristics of excluded cases (n = 89)

**Variable**	**Baseline**
Male*	81(91)

AGE (years)^#^	45.16 (6.80)

IMT (mm)^#^	0.79 (0.26)

FMD (%)^#^	4.67 (4.36)

SBP (mm Hg)^#^	120.8 (15.6)

Total: HDL Cholesterol^#^	5.04 (1.18)

Current smoking status* 1	36 (40.9)

Current STATIN use* 1	9 (10.1)

CD4 Count^#^	451.14 (275.51)

Log_10 _Viral Load^#^	2.4 (1.3)

NB) 1 = current smoker/user; * = count(%); # = mean(standard deviation)

Examining the data cross-sectionally at baseline and follow-up, there was a *dose-response *relationship between carotid IMT and risk group classification (Table 5). Carotid IMT differed significantly by risk group classification at baseline and follow-up (p < 0.001 respectively in each case). There was neither a *dose-response *relationship nor significant difference in brachial FMD across risk groups at baseline and follow-up (p = 0.540 and 0.312 respectively).

**Table 5:** Baseline and follow-up characteristics for progression data by Framingham risk group

**Risk group**	**Number of subjects**	**IMT 1 (Baseline)**	**IMT 2 (Follow-up)**	**FMD 1 (Baseline)**	**FMD 2 (Follow-up)**
Very low (< 5%)	54	0.70 (0.14)	0.72 (0.15)	5.67 (5.88)	4.35 (4.36)

Low (5 to 9%)	46	0.78 (0.17)	0.78 (0.17)	4.83 (3.54)	5.29 (5.13)

Medium/High (10% and above)	68	0.94 (0.24)	0.97 (0.25)	4.83 (4.02)	3.84 (5.27)

NB) Entries for IMT and FMD are reported as mean(standard deviation); Reported to two decimal places.

### Validity of baseline extent measures (cross-sectional data)

Goodness-of-fit tests were satisfied. The distribution of residuals did not deviate systematically from the normal distribution. Validity of measurement method was assessed by how well each method correlated with classical cardiovascular risk factors at baseline. From multiple regression models: older patients (p < 0.001), male patients (p = 0.034), current smokers (p < 0.001), patients with higher SBP (p < 0.001), or higher total:HDL cholesterol (p = 0.004) were statistically significantly associated with higher carotid IMT (Table 6). The cardiovascular risk factors explained approximately 45% of the variation in carotid IMT (R^2 ^= 0.45). Neither current statin use nor CD4 count were statistically significantly associated with IMT (p = 0.904 and 0.929 respectively).

**Table 6:** Estimates from multiple regression models for baseline of Carotid IMT and Brachial FMD (%)

	**CAROTID IMT**			**BRACHIAL FMD**		
**PARAMETER**	**Est**.*	**95% CI**	**p-value**	**Est**.*	**95% CI**	**p-value**

Age (years)	0.016	(0.014, 0.019)	< 0.001	-0.021	(-0.093, 0.051)	0.569

Male	0.081	(0.006, 0.155)	0.034	-1.738	(-3.601, 0.124)	0.067

Current smoking status	0.096	(0.050, 0.143)	< 0.001	0.294	(-0.874, 1.462)	0.620

SBP (mm Hg)	0.003	(0.002, 0.005)	< 0.001	-0.021	(-0.058, 0.016)	0.262

Total:HDL Cholesterol	0.026	(0.008, 0.043)	0.004	0.001	(-0.435, 0.438)	0.995

Current STATIN use	-0.006	(-0.096, 0.085)	0.904	1.578	(-0.683, 3.839)	0.171

CD4 Count	-0.000004	(-0.00009, 0.00008)	0.929	-0.001	(-0.003, 0.001)	0.512

NB) *Est. – Estimate.

In contradistinction, none of these risk factors was significantly associated with brachial FMD (Table 6). The cardiovascular risk factors explained only 3% of the variation in brachial FMD (R^2 ^= 0.031). Current use of statins explained negligible amount of variation in both IMT and FMD regression models. It should however be noted that the percentage of patients on statin was very small to make strong inferences regarding the effect of the drug.

### Responsiveness to change (progression data)

The "continuous time" AR(1) structure was assumed for carotid IMT while the "fixed time" structure was assumed for brachial FMD using results from the AICs. Both models provided better fits than the independent correlation structure model from the "Null Model Likelihood Ratio" tests.

From fixed-effects models, positive change in carotid IMT was statistically significantly associated with older age (p < 0.001), male gender (p = 0.005), and current smoking status (p = 0.011). Increase in SBP or total:HDL cholesterol was not statistically significantly associated with progression of carotid IMT (Table 7).

**Table 7:** Estimates from fixed effects models for progression of Carotid IMT and Brachial FMD (%)

	**CAROTID IMT**			**BRACHIAL FMD**		
**PARAMETER**	**Est**.*	**95% CI**	**p-value**	**Est**.*	**95% CI**	**p-value**

Time (years)	0.001234	(-0.01556, 0.01803)	0.8847	0.7342	(-0.2578, 1.7261)	0.1457

Age (years)	0.01550	(0.01235, 0.01865)	< .0001	0.02485	(-0.04543, 0.09513)	0.4857

Male	0.1225	(0.03721, 0.2078)	0.0051	-0.1125	(-2.0420, 1.8169)	0.9085

Current smoking status	0.07073	(0.01658, 0.1249)	0.0108	-1.1385	(-2.3578, 0.08092)	0.0671

SBP	0.000726	(-0.00028, 0.001730)	0.1544	-0.02425	(-0.06244, 0.01395)	0.2116

Total:HDL Cholesterol	0.01051	(-0.00392, 0.02494)	0.1520	-0.2449	(-0.6936, 0.2038)	0.2824

Current STATIN use	0.06222	(-0.05335, 0.1778)	0.2893	3.1025	(0.5174, 5.6876)	0.0190

CD4 Count	0.0000009	(-0.00002, 0.000020)	0.9265	0.000085	(-0.00075, 0.000924)	0.8411

NB) *Est. – Estimate.

In comparison to non-statin users, patients on current (baseline) statin medication had significantly better FMD response after one-year follow-up (mean difference = 3.11, 95% CI: 0.53 to 5.69). None of the traditional cardiovascular risk factors was significantly associated with progression of brachial FMD (Table 7).

## Discussion

Non-invasive, validated and reproducible arterial imaging techniques such as brachial FMD and carotid IMT are often used to measure the extent, severity or progression of subclinical atherosclerosis in vascular health studies [13,20]. Brachial FMD is a measure of endothelial dysfunction [13,20] whereas carotid IMT measures structural vascular integrity [13]. Studies have shown that anti-atherogenic interventions such as statins, angiotensin-converting enzyme (ACE) inhibitors and other blood-pressure lowering agents help to improve brachial FMD [13,32,39,40], and retard carotid IMT progression [12,13,30,31], thus highlighting the importance of both measures in the atherosclerotic process.

In our study of HIV patients, neither extent nor progression of brachial FMD was significantly associated with any of the examined classical vascular risk factors. The cardiovascular risk factors explained only 3% of the variation in brachial FMD. Use of statin medication led to statistically significant improvement in brachial FMD, thus replicating results from other studies [39]. Extent of carotid IMT was significantly associated with age, male gender, current smoking status, SBP and total:HDL cholesterol, whereas progression of carotid IMT was significantly associated with age, male gender and current smoking status. The cardiovascular risk factors explained approximately 45% of the variation in carotid IMT.

Our results on carotid IMT are similar to results obtained in other vascular studies in both non-HIV [13,15,41,42] and HIV subject populations [1,43]. In a cross-sectional study involving 119 indigenous Australians at risk of cardiovascular disease, carotid IMT was significantly associated with traditional cardiovascular risk factors, while brachial FMD was associated with none of the examined risk factors [28]. A case-control study by Lekakis et al [3] found a significant association between extent of IMT and blood pressure, cholesterol and glucose levels, duration of HIV disease and use of protease inhibitors. In contrast, brachial FMD was only associated with triglyceride measurements [3]. Yan et al [13] in a cross-sectional analysis of data from a large cohort of middle-aged healthy men found significant associations between carotid IMT increase and age, SBP, body mass index, total and LDL cholesterol and fasting plasma glucose. Among all risk factors examined, increasing SBP was the only one associated with impaired brachial FMD [13]. In a small study (total sample size = 37) involving a relatively homogenous sample of adult HIV patients on anti-retroviral therapy, Stein et al [44] found an association between impaired brachial FMD and VLDL (very low density), IDL (intermediate density), HDL and total cholesterol levels [44]. Brachial FMD has been shown to correlate with vascular risk factors in non-HIV subjects [24,27] and use of protease inhibitors in HIV subjects [44].

We also found a weak inverse relationship between carotid IMT and brachial FMD with borderline significance (r = -0.126, p = 0.043). A much larger study (sample size of 1,578) by Yan et al [11] found no significant correlation (r = -0.006, p = 0.82) between IMT and FMD in healthy middle-aged men without cardiovascular disease [11]. Irace et al [45] found a moderate linear association between FMD and IMT in treatment naïve subjects at risk of CAD (r = -0.217, p = 0.058). In a large study involving 2,109 healthy adults aged 24 to 39 years in Finland, Juonala et al [46] found a statistically significant inverse relationship (p < 0.001) between IMT and FMD, thus adding to a series of conflicting results on the "true" nature of the relationship between these two important measures. Several relatively smaller studies have found significant inverse relationship between IMT and FMD suggesting that these two measures assess the same "aspects and stages of early atherosclerosis" [47-52]. The results from smaller studies are suspect due to sample size limitation. Findings from Yan et al [13] suggest that brachial FMD and carotid IMT are likely "unique" and unrelated surrogates that assess varying aspects and stages vascular disease [13]. In contrast, Juonala et al [46] suggest a strong inverse relationship between FMD and IMT, which would be expected if both measures are assessing the same construct. However, we note that while Yan et al [13] employed an IMT method that includes both far and near walls of all segments in the right and left carotid arteries (similar to our study), Juonala et al [46] employed a method that includes only the far wall of the left carotid artery. Perhaps this may serve to explain the contrasting results.

Various explanations have been proposed for conflicting results regarding brachial FMD in the literature. These include heterogeneity in patient populations being studied, different measurement protocols or inadequate sample sizes [11,13,14]. In our study, brachial FMD was measured using an extensively validated and reliable method [13,31-33]. Rundek et al [11] suggest a possibly *direct *relationship between endothelial dysfunction and atherosclerosis, independent of traditional vascular risk factors. Thus beyond traditional vascular factors, endothelial dysfunction may independently provide additional prognostic information on atherosclerosis through other *risk factors *not currently assessed [11,13,20]. Nevertheless, the validity of brachial FMD as a measure of cardiovascular risk in HIV remains largely unproven. There is need for large, long-term observational studies (with standardized FMD protocols) to critically evaluate the specific role of brachial FMD in atherosclerosis relating to HIV patients. The results presented in our paper were based on baseline and one-year follow-up results.

From our study, IMT progressed at an annual rate of 0.02 mm/year. Hsue et al [1] estimated the annual progression of IMT as 0.074 mm/year in an ancillary cohort study involving 121 HIV-infected adults [1]. The distinction between progression estimates from different studies may result from demographic or clinical differences in the HIV populations studied. Further, more precise progression estimates can be obtained from studies with longer follow-up such as the ongoing "Canadian HIV vascular study". The Canadian HIV vascular study also aims to investigate the relationship between atherosclerotic progression, anti-retroviral drug regimen and immune reconstitution.

There were significant cross-sectional *dose-response *relationships between baseline (or follow-up) carotid IMT and Framingham risk group classification. Framingham risk classification was a strong predictor of extent of carotid IMT, thus highlighting the prognostic value of risk group classification.

The use of fixed effects models to analyze progression data is one of the strengths of our study. Fixed effects models allow for the inclusion of time-varying covariates such as age, SBP and total:HDL cholesterol. Changes in these covariates are likely to affect progression of either brachial FMD or carotid IMT, thus including this information in model specification is vital to obtaining a closer representation of reality. Secondly, the use of the "continuous-time autoregressive correlation structure" option in SAS software allowed for patients to have differential follow-up times, which more closely depicts circumstances surrounding our study. Also, information on the correlation between baseline and follow-up outcome measures was included as part of model specification.

## Conclusion

Carotid IMT is a useful surrogate marker of extent and progression of cardiovascular risk in HIV patients 35 years of age and older, correlating better than FMD with established cardiovascular risk factors. Extent of carotid IMT correlates well with current risk stratification of patients using Framingham risk scores. Use of carotid IMT in ongoing and future observational studies and randomized trials may help to better define the atherosclerotic risk associated with HIV infection and with specific HIV treatments.

Comparison of results across studies is often quite difficult due to differing measurement protocols employed by different investigators. Standardization of protocols for FMD and IMT will aid the comparison of results across studies.

## Competing interests

MS has investigator-initiated grant support from Gilead Sciences and Pfizer. LT consults with GlaxoSmithKline Inc. (GSK) on statistical and other methodological issues. No other potential conflicts to report.

## Authors' contributions

AO wrote data analysis plan, conducted data analysis and wrote the first draft of manuscript with inputs from MS and LT. MS is principal investigator on HIV vascular cohort study. MS, LT, FS, KG, JG, TA, DE, SS, JB, EL and AO made substantial contributions to manuscript content through subsequent drafts. MS, FS, KG, JG, TA, DE, SS and EL participated in data collection at the various centers. All authors read and approved the final manuscript.
